# Supplementary material for: Predicting visual function by interpreting a neuronal wiring diagram
Source: Nature. 2024 Oct 2;634(8032):113–23. doi: 10.1038/s41586-024-07953-5 (PMC11446822; doi:10.1038/s41586-024-07953-5)
Supplement: Supplementary file 6 — CRF and ERF predictions for individual TmY4 and TmY9 cells. Analogous to Supplementary Data 3, but for TmY target types. Shown are the top four monosynaptic pathways, the strongest pathway passing through each of the top ten intermediary types (ranking from Extended Data Fig. 7), and the trisynaptic pathway Tm1–TmY–Dm3–TmY (see the section entitled Prediction of spatial normalization). [file 41586_2024_7953_MOESM6_ESM.zip › DataS4/TmY9qΓèÑ/720575940613999510.pdf]

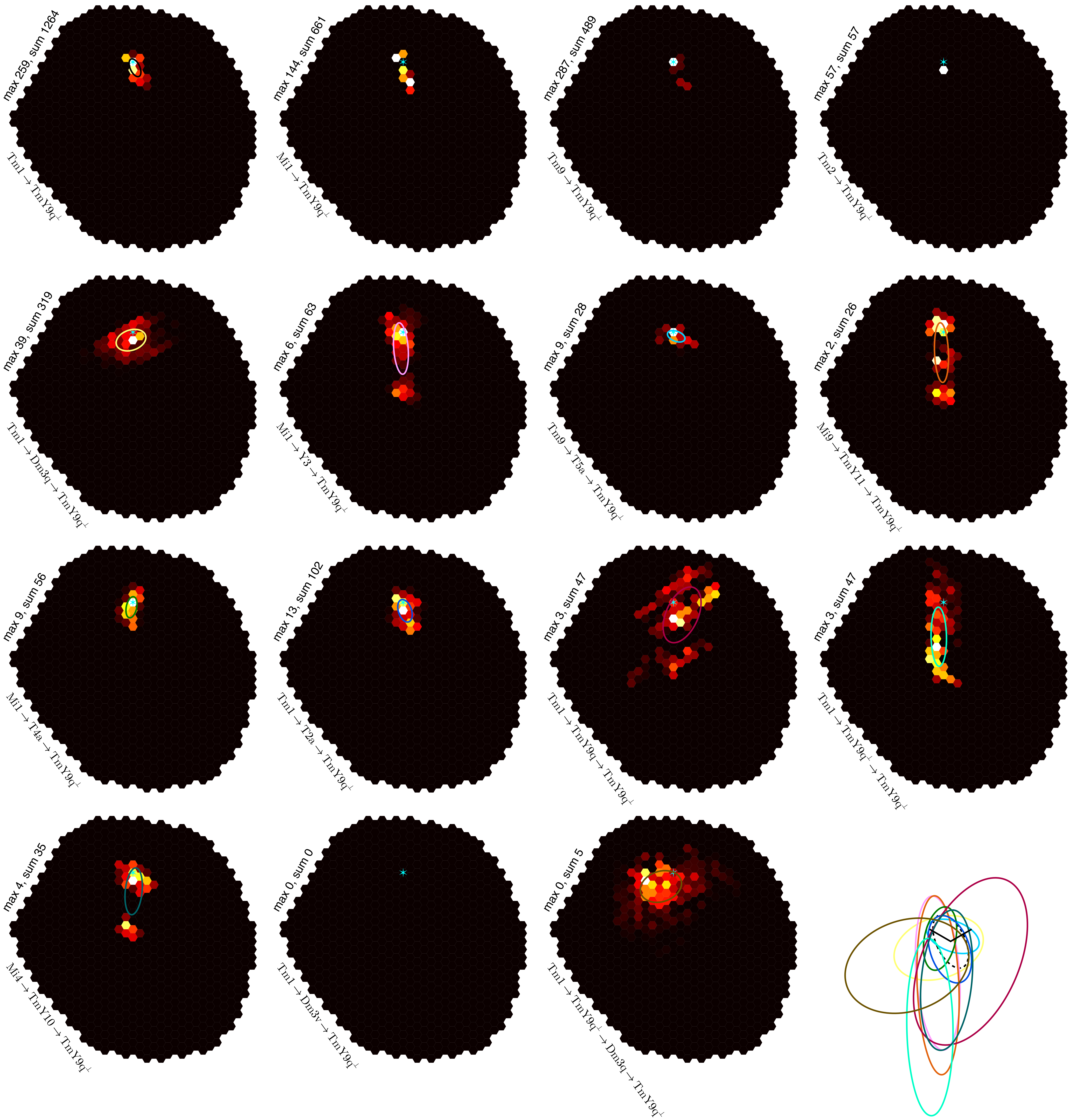
max 259, sum 1264
Tm1 → TmY9q⊥
max 144, sum 661
Mi1 → TmY9q⊥
max 287, sum 489
Tm9 → TmY9q⊥
max 57, sum 57
Tm2 → TmY9q⊥
max 39, sum 319
Tm1 → Dm3q → TmY9q⊥
max 6, sum 63
Mi1 → Y3 → TmY9q⊥
max 9, sum 28
Tm9 → T5a → TmY9q⊥
max 2, sum 26
Mi9 → TmY11 → TmY9q⊥
max 9, sum 56
Mi1 → T4a → TmY9q⊥
max 13, sum 102
Tm1 → T2a → TmY9q⊥
max 3, sum 47
Tm1 → TmY9q → TmY9q⊥
max 3, sum 47
Tm1 → TmY9q⊥ → TmY9q⊥
max 4, sum 35
Mi4 → TmY10 → TmY9q⊥
max 0, sum 0
Tm1 → Dm3v → TmY9q⊥
max 0, sum 5
Tm1 → TmY9q⊥ → Dm3q → TmY9q⊥
